# A Novel Approach for Mixed Manual/Connected Automated Freeway Traffic Management

**DOI:** 10.3390/s20061757

**Published:** 2020-03-22

**Authors:** Duo Li, Peter Wagner

**Affiliations:** 1 School of Highway, Chang’an University, Xi’an 710064, China; 2 Institute of Transport Systems, German Aerospace Center (DLR), 12489 Berlin, Germany; Peter.Wagner@dlr.de

**Keywords:** connected vehicle, freeway traffic control, type-2 fuzzy logic

## Abstract

Freeway traffic management and control often rely on input from fixed-point sensors. A sufficiently high sensor density is required to ensure data reliability and accuracy, which results in high installation and maintenance costs. Moreover, fixed-point sensors encounter difficulties to provide spatiotemporally and wide-ranging information due to the limited observable area. This research exploits the utilization of connected automated vehicles (CAVs) as an alternative data source for freeway traffic management. To handle inherent uncertainty associated with CAV data, we develop an interval type 2 fuzzy logic-based variable speed limit (VSL) system for mixed traffic. The simulation results demonstrate that when more 10% CAVs are deployed, the performance of the proposed CAV-based system can approach that of the detector-based system. It is demonstrated in addition that the introduction of CAVs may make VSL obsolete at very high CAV-equipment rates.

## 1. Introduction

In the past half century, various traffic control measures have been developed to improve mobility, safety and environmental performance of freeway systems. Among them, variable speed limit (VSL) is one of the most commonly applied tools to achieve such a goal. The objectives of VSL are the harmonization of traffic flows and improvement in efficiency and safety by advising/forcing drivers to adjust their speeds to better respond to prevailing traffic conditions. 

To determine proper speed limit values, conventional VSL systems use traffic data collected from fixed-point sensors, such as inductive loops. Such an observation method requires a sufficiently high sensor density (e.g., 500 m spatial resolution) to ensure data reliability and accuracy. Nevertheless, installation and maintenance of fixed-point sensors entail considerable cost; this might be the main reason behind sparse application of advanced traffic control systems around the world. Moreover, although fixed-point sensors can acquire detailed information at devices’ installed points, they encounter difficulties to provide spatiotemporally and wide-ranging information due to the limited observable area [1]. There is a need, therefore, to exploit different, less costly data sources for VSL systems.

Recent achievements in connected automated vehicle (CAV) technology provides great potential for addressing the limitations associated with existing VSL systems. The introduction of CAVs may bring several benefits to traffic control systems. First, as mobile sensors, CAVs can provide wide-ranging and spatiotemporally detailed information, because CAVs can range over the entire road network. Second, applying CAVs can improve mobility, safety and environmental performance of existing traffic infrastructures [1]. 

With the promising role that CAV technology can play in the next generation of traffic systems, a substantial body of literature has exploited the utilization of CAVs as an alternative data source. In [2,3,4,5,6], the traffic state was estimated based on vehicle trajectory data. Although the majority of existing studies focus on the traffic state and travel time estimation, a few attempts have been made on the further exploitation of CAVs for freeway traffic control and traffic signal control [7,8]. Utilizing CAVs rather than fixed-point sensors as the data source is expected to reduce the overall cost associated with the implementation of advanced traffic control systems. Such “infrastructure-free” systems can provide a cost-effective solution, especially for jurisdictions with a lack of resources for installation of traffic monitoring infrastructure. This research presents a novel approach for “infrastructure-free” freeway traffic control. The presented approach uses CAV data as control input and is designed based on a new class of fuzzy logic—interval type 2 fuzzy logic.

## 2. Literature Review

The VSL literature can be broadly classified in two groups, namely reactive rule-based algorithms and proactive algorithms. Rule-based VSL algorithms generally apply thresholds for volume [9], occupancy/density [10], speed [11] or their combinations [12] to determine speed limit values. A VSL using fuzzy logic control [13] is a special case of this type of control algorithm. Most of the existing VSL systems use rule-based algorithms in practice. Proactive algorithms were developed to enhance the performance of rule-based ones. In this category of VSL algorithms, future traffic is predicted with consideration of complex traffic dynamics. Model predictive control (MPC) based VSL proposed by Hegyi et al. [14] is the most prominent example of proactive algorithms, which aims to suppress shockwaves in a metastable state traffic condition. They modified a general second-order traffic flow model—modéle d’écoulement de trafic sur autoroute networks (METANET) to incorporate the effect of VSL into the calculation logic. The advantages of the proactive approach have been made clear through its adoption in several subsequent studies [15,16]. 

As discussed in the previous section, most of the VSL systems are based on traffic data obtained from fixed point sensors. There are a limited number of studies aimed at [4] proposing a candidate VSL algorithm that uses space mean speeds (SMSs) collected from on-vehicle devices as its main input. The proposed algorithm extended the capability of an MPC based VSL with the input of SMSs and their corresponding space-based densities, rather than spot-based speeds and densities. The authors stated that the developed probe-based VSL algorithm was comparable to its detector-based counterpart under a relatively high probe vehicle share. Khondaker and Kattan [17] proposed a VSL control algorithm for simultaneously minimizing the total travel time (TTT), time to collision (TTC) and fuel consumption in a connected vehicle environment. The authors used the position, speed and acceleration of each connected vehicle as the input. They reported that the gains of the proposed algorithm were insignificant in terms of mobility, safety and sustainability.

Previous papers leave several issues that could be enhanced. Firstly, in the aforementioned studies, traffic states (e.g., density) derived from CAV data were directly used as inputs for VSL controllers. However, estimated traffic states are unavoidably associated with estimation errors. Under/overestimated traffic states may result in imprecise control actions. Secondly, the majority of the proposed CAV-based VSL systems still partially rely on the information from fixed point sensors. There are two major contributions of this work. First, to the best of our knowledge, this is the first work that takes into account estimation errors in freeway traffic control process. Second, this work presents an “infrastructure-free” system that is completely based on the information from CAVs. 

## 3. Methodology

**Figure 1:**
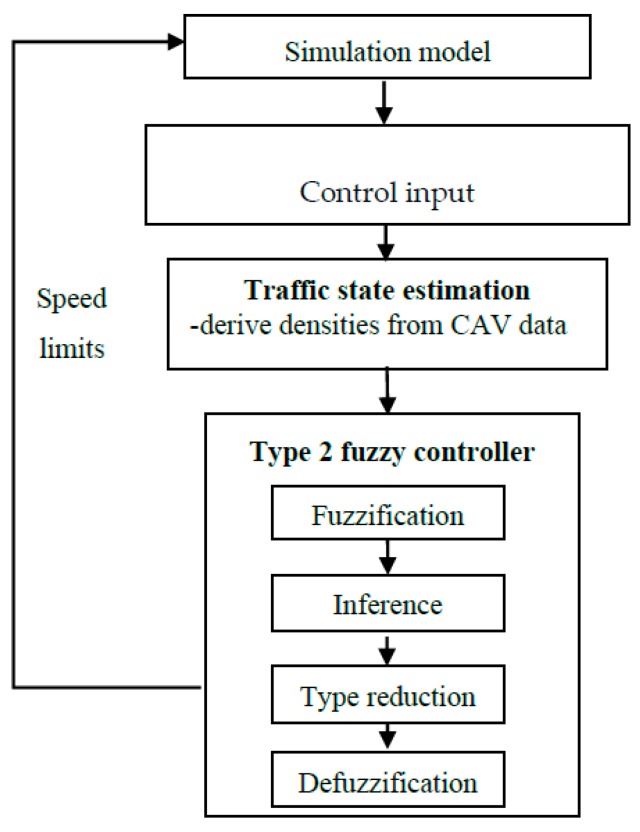
Interval type-2 fuzzy based variable speed limit (VSL) control framework.

Figure 1 illustrates the interval type-2 fuzzy logic control framework followed for the developed CAV-based VSL approach. The framework consists of three major interrelated components: (1) data input, (2) traffic state estimation and (3) interval type-2 fuzzy logic control. At each time step, speed and position data are collected from CAVs. Next, traffic state (e.g., density) is estimated on the basis of collected CAV data. Then, the type-2 fuzzy control process is performed to determine appropriate speed limit values for current traffic conditions. Finally, control actions are applied to the traffic flow. 

The following conditions are assumed for traffic in this study: For each inserted vehicle, it is decided randomly whether it is a CAV or not, with the probability equipment rate. CAVs are equipped with global navigation satellite system (GNSS) devices that collect data, including position and speed. The average speed of all vehicles at a freeway segment equals the average speed of CAVs at the same segment. This assumption has been validated by previous studies [18]. The following subsections describe the details of the proposed approach.

### 3.1. Traffic State Estimation

A fundamental diagram (FD) describes the relationship between traffic speed *u*, density *k* and flow *q.* In this study, we utilize the speed–density relationship, i.e., estimate *k* for a given *v*, because the typical CAV data only consists of speed and position of the vehicle itself. In previous studies, various single regime models have been proposed to describe the speed–density relationship, such as Greenshields [19], Underwood [20] and Van Aerde [21]. In this research, we employ the Van Aerde model, which combines the macroscopic and microscopic view on traffic flow. The functional form of the model combines the Greenshields and Pipes car-following models, as shown in Equations (1)–(4). This form provides an additional degree of freedom by enabling the speed at capacity *v_c_* to be different from free-flow speed *v_f_*. The Van Aerde model overcomes the weaknesses of the Pipes model that assumes *v* is insensitive to *k* in the uncongested regime and the Greenshields model in which the speed–flow relationship is parabolic.

(1) $$k=\frac{1}{c_{1}+\frac{c_{2}}{v_{f}+v}+c_{3}v}$$

(2) $$m=\frac{2v_{c}-v_{f}}{{\left(v_{c}-v_{f}\right)}^{2}}$$

(3) $$c_{2}=\frac{1}{k_{j}\left(m+\frac{1}{v_{f}}\right)}$$

(4) $$c_{1}=mc_{2}$$

(5) $$c_{3}=\frac{-c_{1}+\frac{v_{c}}{q_{c}}-\frac{c_{2}}{v_{f}-v_{c}}}{v_{c}}$$

Here, *c*_1_ is the fixed distance headway constant; *c*_2_ is the first variable distance headway constant; *c*_3_ is the second variable distance headway constant; *v_f_* is free flow speed; *v_c_* is speed at capacity; *q_c_* is flow at capacity; *k_j_* is jam density; and *m* is a constant used to solve for the three headway constants.

Before being used for traffic state estimation, the model needs to be calibrated so as to have a model for the study area at hand. As shown in Equations (1)–(4), the calibration of the model requires estimating four model parameters, namely *v_f_*, *v_c_*, *q_c_* and *k_j_*. The model calibration is performed by minimizing the following objective function, which measures the difference between the data coming from the Van Aerde model, depending on the parameters to calibrate, and the “real” data coming from the simulation model:

(6) $$J=\sqrt{[\overset{n}{\underset{i=1}{\sum }}{\left(F_{i}-O_{i}\right)}^{2}]/n}$$

where *F_i_* is the *i*th data point from the Van Aerde model; *O_i_* is the *i*th data point from the simulation model; and *n* is the number of samples. We modelled the selected freeway section using traffic data collected from 5 to 6 pm of 10 weekdays; 600 data points (10 days*60 min) including speed and density were collected from the simulation model and used as the “real” data points (*O_i_*) in the calibration process. Sequence quadratic programming (SQP) is used to solve the optimization problem. The SQP is computed using the *fmincon* function of the optimization toolbox of MATLAB.

### 3.2. Interval Type-2 Fuzzy Logic Control

Although the aforementioned Van Aerde model has been carefully calibrated, we found that there still existed certain errors within estimation results. These uncertainties may result in over/under-estimated density values and consequently degrade the effectiveness of VSL systems due to inaccurate control inputs.

In this paper, we introduce a class of fuzzy logic control systems—type-2 fuzzy logic system (T2-FLS)—which has not been used in traffic control so far. It has the ability to handle uncertainties within the estimated densities. The concept of a T2-FLS was introduced by Zadeh [22] as an extension of an ordinary fuzzy logic system (henceforth called a type-1 fuzzy logic system or T1-FLS for short). A T2-FLS is characterized by a fuzzy membership function (MF), i.e., the membership value for every element is a fuzzy set in [0,1], where the membership value of a T1-FLS is a crisp point between 0 and 1. There are several sources of uncertainties in T1-FLSs, e.g., noisy measurements and linguistic uncertainties [23]. T1-FLSs are unable to deal with such uncertainties since their MFs (two-dimensional) are totally crisp, while T2-FLSs can handle these uncertainties as their MFs (three-dimensional) are themselves fuzzy. Hisdal [24] stated that increasing the fuzziness of a description by applying a higher type of FLSs can result in the increased ability to deal with inexact information in a logically correct manner. Therefore, the features of T2-FLSs make them particularly suitable for traffic control systems with noisy measurements, i.e., VSL systems with estimated densities in our case. 

Due to the computational complexity of a general T2-FLS, the majority of the researchers use a special case of the general T2-FLS, namely interval T2-FLS (IT2-FLS) [25]. Manageable computations associated with IT2-FLSs makes them more practical when compared against the general T2-FLS. Thus, in the rest of the paper we are only interested in IT2-FLSs. 

**Figure 2:**
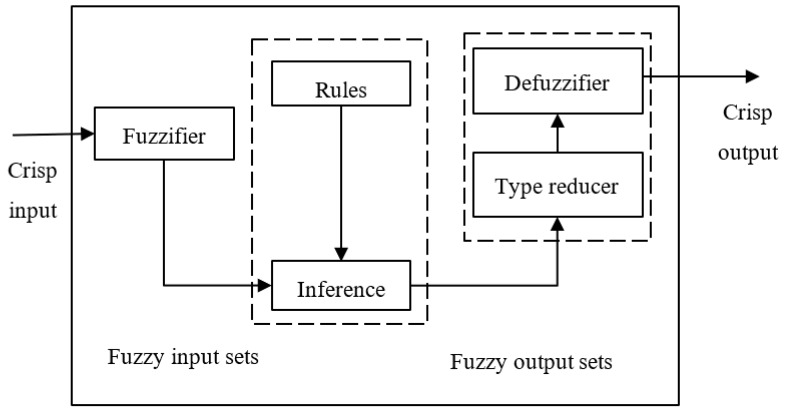
Schematic diagram of IT2 FLS.

The schematic diagram of an IT2-FLS is depicted in Figure 2, which is very similar to its T1 counterpart. The major difference is that IT2-FLS needs a type-reducer to transfer IT2-FSs into T1-FSs before defuzzification can be carried out. To construct an IT2-FLS based VSL controller, we use the density derived from CAV data as the input and the speed limit as the output.

**Figure 3:**
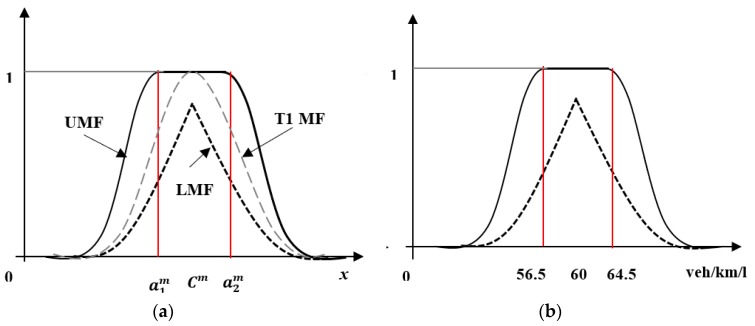
(**a**) Gaussian IT2 MF and (**b**) its example.

This study employs two forms of IT2 MFs, namely, a Gaussian MF and a triangular MF. The case of a Gaussian primary MF $\mu^{m}$ (see Figure 3a) with an uncertain mean *a^m^* varying between $\left[a_{1}^{m},a_{2}^{m}\right]$ and a fixed standard deviation $\sigma^{m}\;$can be expressed as:

(7) $$\mu^{m}\left(x\right)=\exp\left[-\frac{1}{2}{\left(\frac{x-a^{m}}{\sigma^{m}}\right)}^{2}\right],\;\;a^{m}\;\in \left[a_{1}^{m},a_{2}^{m}\right]$$

(8) $$a_{1}^{m}=C^{m}+a^{\prime }-\sigma^{\prime }$$

(9) $$a_{2}^{m}=C^{m}+a^{\prime }+\sigma^{\prime }$$

where, *C^m^* is the centroid of the corresponding T1 MF;$\;a^{\prime }\mathrm{and}\;\sigma^{\prime }$ are the mean and standard deviation of estimation errors respectively.

The upper membership function (UM) ${\bar{\mu }}^{m}\left(x\right)$ is given by

(10) $${\bar{\mu }}^{m}\left(x\right)=\{\begin{array}{ll} N\left(a_{1}^{m},\;\sigma^{m};x\right) & x<a_{1}^{m}\\ 1 & a_{1}^{m}\leq x\leq a_{2}^{m}\\ N\left(a_{2}^{m},\;\sigma^{m};x\right) & x>a_{2}^{m} \end{array}$$

where, for example,

(11) $$N\left(a_{1}^{m},\;\sigma^{m};x\right)≜\exp\left[-\frac{1}{2}{\left(\frac{x-a_{1}^{m}}{\sigma^{m}}\right)}^{2}\right]$$

The lower membership function (LM) ${\underline{\mu }}^{m}\left(x\right)$ is given by

(12) $${\bar{\mu }}^{m}\left(x\right)=\{\begin{array}{ll} N\left(a_{1}^{m},\;\sigma^{m};x\right) & x>\frac{a_{1}^{m}+a_{2}^{m}}{2}\\ N\left(a_{2}^{m},\;\sigma^{m};x\right) & x\leq \frac{a_{1}^{m}+a_{2}^{m}}{2} \end{array}$$

An example of a Gaussian IT2 MF is shown in Figure 3b. In this example, the mean $a^{\prime }$ and standard deviation $\sigma^{\prime }$ of errors associated with density estimation are 0.5 veh/km/ln and 4 veh/km/ln respectively and the centroid of the corresponding T1 MF is 60 veh/km/ln. Thus, the uncertain mean *a^m^* ranges between $\;a{\;}_{1}^{m}\;$= 60 + 0.5 − 4 = 56.5 veh/km/ln and $\;a{\;}_{2}^{m}$= 60 + 0.5 + 4 = 64.5 veh/km/ln.

**Figure 4:**
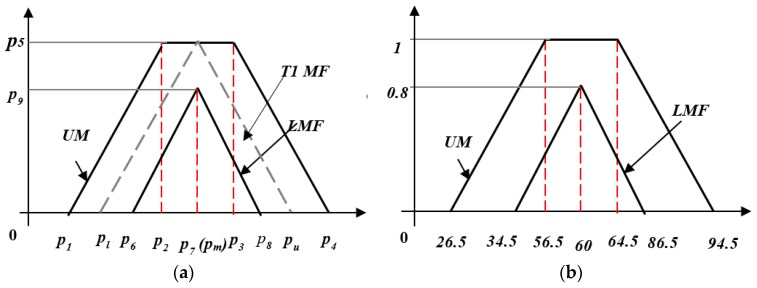
(**a**) Triangular IT2 MF and (**b**) its example.

A triangular IT2 MF is defined by a 9-point vector *P* = (*p*_1_, …, *p*_9_), as illustrated in Figure 4a. Given a triangular T1 MF defined by a lower limit *p_l_*, an upper limit *p_u_*, and a centroid *p_c_*, its IT2 counterpart can be expressed as

(13) $$p=\left[\begin{array}{c} p_{1}=p_{l}+a^{\prime }-\sigma^{\prime },\\ p_{2}=p_{c}+a^{\prime }-\sigma^{\prime },\\ p_{3}=p_{c}+a^{\prime }+\sigma^{\prime },\\ p_{4}=p_{u}+a^{\prime }-\sigma^{\prime },\\ p_{5}=1,\\ p_{6}=p_{l}+a^{\prime }+\sigma^{\prime },\\ p_{7}=p_{c},\\ p_{8}=p_{u}+a^{\prime }-\sigma^{\prime },\\ p_{9}=(p_{c}-a^{\prime }-\sigma^{\prime })/\left(p_{c}-p_{l}\right), \end{array}\right]$$

Figure 4b presents an example of a triangular IT2 MF in which the $a^{\prime }$ and $\sigma^{\prime }$ values are the same to the previous Gaussian MF example. For a given triangular T1 MF defined by (30, 60, 90), its IT2 counterpart is represented as (26.5, 56.5, 64.5, 94.5, 1, 34.5, 60, 86.5, 0.85).

**Figure 5:**
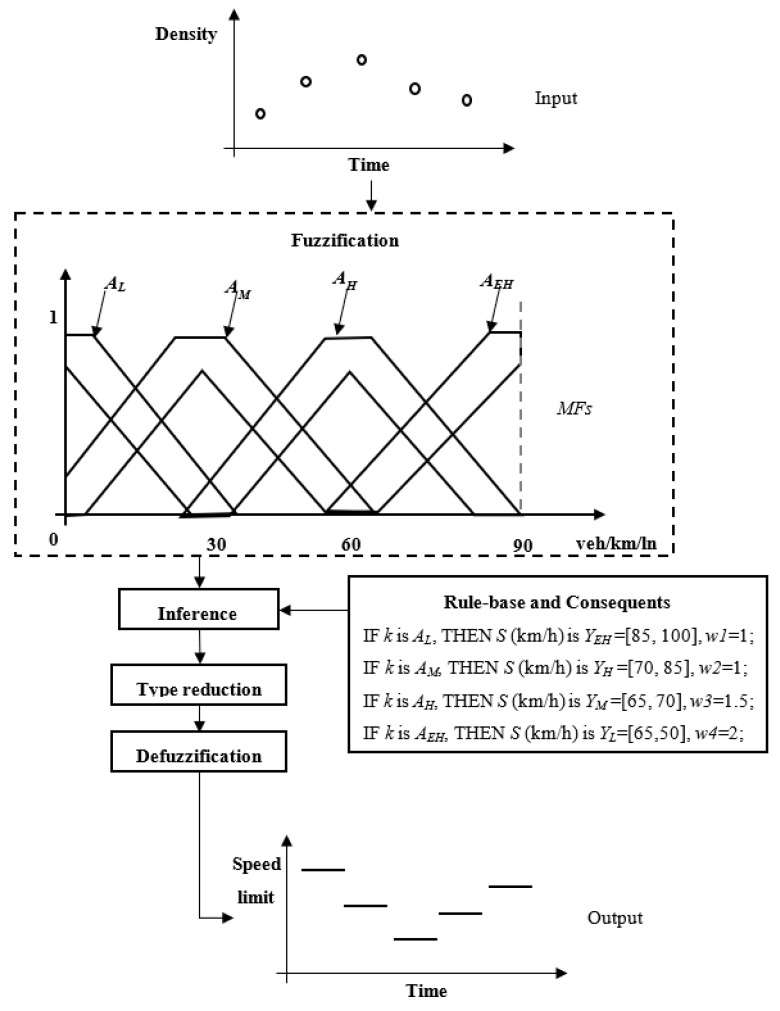
An example of IT2 FLS settings.

The rule-base and the corresponding consequents of the proposed IT2 FLS are illustrated in Table 1. And Figure 5 shows an example of the proposed IT2-FLS settings.

## 4. Simulation Experiment

**Figure 6:**
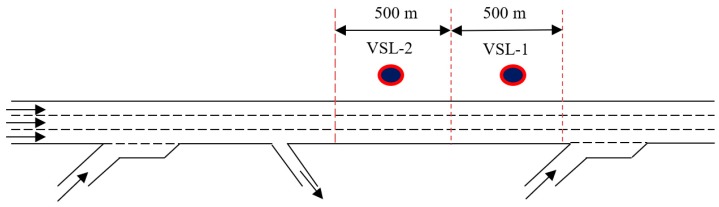
Layout of the test bed.

The effectiveness of the proposed VSL algorithm was verified against a real freeway section in Auckland, New Zealand (see Figure 6). The chosen test bed consists of two on-ramps and one off-ramp. The test bed was simulated using the Simulation of Urban MObility (SUMO) micro-simulator [26] for the following reasons. Firstly, SUMO is open-source. Secondly, SUMO has a flexible architecture and can be enhanced with custom models. Thirdly, SUMO has a track record of research behind it. The network data used in this study was provided by New Zealand Transport Agency (NZTA), which includes loop detector measurements from the on-ramps, off-ramps and mainline accumulated over a 30 second time period. Ten weekdays with fine weather condition and typical traffic demand were chosen from 5 March to 27 May 2012 for the purpose of simulation experiments. The simulation model was calibrated and validated based on the Geoffrey E. Havers (GEH) index [27]. GEH index is a commonly used criterion for model calibration and validation: where E is estimated count using the SUMO model, and F is the field count. Five-minute vehicle counts from the simulation outputs and real world measurements were used to compute GEH values of 9 different locations. For all of the selected weekdays, at least 8 out of 9 locations produced GEH values that were smaller than 5. Therefore, the developed model is considered to be acceptable.

The IT2-FLS based VSL control was realized via *IT2FLS* toolbox developed by Taskin and Kumbasar [28] in MATLAB. The communication between MATLAB and SUMO was created by using *TraCI4Matlab* [29], which is an API allowing the interface between SUMO and any application developed in MATLAB.

The proposed CAV-based VSL was assessed with two different data sources, namely, probe vehicles (PVs) and CAVs. The major difference between PVs and CAVs is their “driver behaviors”. A CAV is assumed to have a smaller headway, a lower driver imperfection and a high compliance rate to speed limits when compared to a human-driven vehicle (HDV). We used the default car following model [30] of SUMO to simulate both types of vehicles. We set the minimum headway time *T* = 0.5 s (CAV)/1.1 s (HDV), the driver imperfection *d_a_* = 0 (CAV)/0.7 (HDV) and the speed factor *d_v_* = 0.01 (CAV)/0.15 (HDV). Note that the lower driver imperfection value (between 0 and 1) of the CAVs leads to more accurate acceleration actions. The speed factor *d_v_* is the coefficient of variance of the desired speed, where the desired speed includes speed-limits. In other words, there is a chance that simulated drivers do not follow the speed-limits exactly but have a certain tolerance.

## 5. Analysis Results

This section presents the simulation results for four different control scenarios, including the following:

- No Control (*NC* for short) scenario, which is used as a baseline to measure improvements offered by other control scenarios;
- T1-FLS based VSL with detector data (*T1D* for short) scenario in which speed limits are determined using T1-FLS and data directly collected from loop detectors;
- T1-FLS based VSL with PVD (*T1P* for short) scenario in which speed limits are determined using T1-FLS and data collected from PVs or CAVs; and
- IT2-FLS based VSL with PVD (*IT2* for short) scenario in which speed limits are determined using the proposed IT2 FLS and data collected from PVs or CAVs.

**Figure 7:**
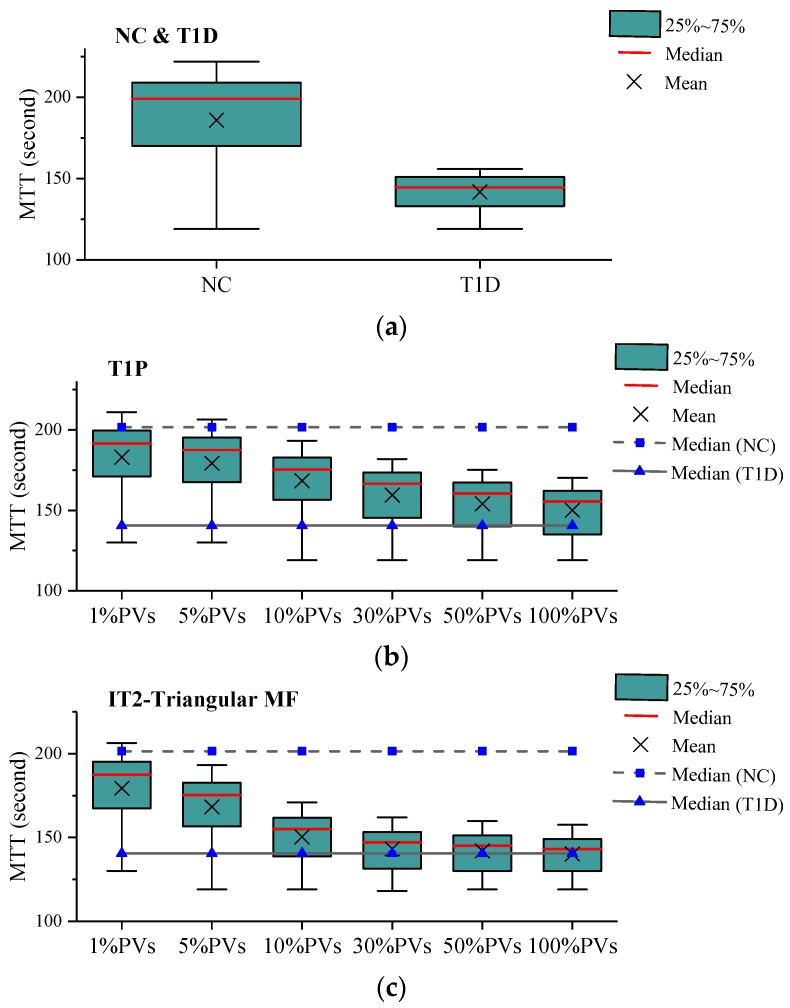

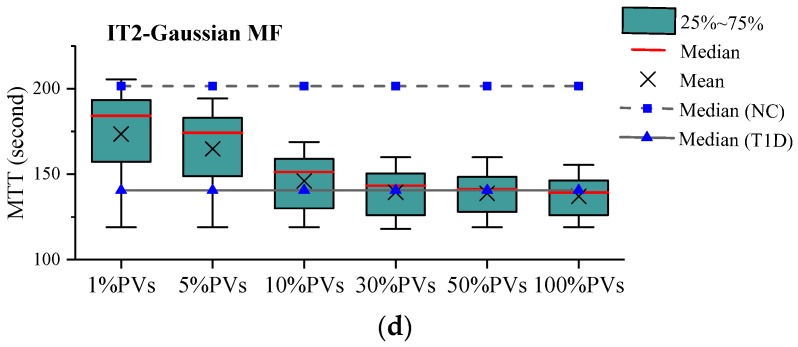
Boxplots of MTT values (the network equipped with PVs): (**a**) NC&T1D scenario, (**b**) T1P scenario, (**c**) IT2-Triangular MF scenario, (**d**) IT2-Gaussion MF scenario.

Mean travel time (MTT) was selected to measure the mobility benefits of the VSL system with different data sources. Figure 7 shows the MTT values computed using 10-day data and the network equipped with PVs. Here, the real traffic data collected from 5–6 pm of the selected weekdays was used to simulate the heavily congested condition. It was observed that the VSL with detector data (T1D) recorded the lowest MTT median and variance among all the tested cases. The IT2 fuzzy-based VSL outperformed its T1 counterpart when data was extracted from PVs. The equipment rate of PVs may affect the effectiveness of the proposed VSL. Several levels of PV equipment rates were thus tested. The simulation results indicated that PV equipment rate played a vital role in the proposed VSL-based control. The performance of the proposed VSL was improved by increasing PV shares. The 100% equipment rate was shown to result in the best performance. Although the IT2 based VSL with Gaussian MFs outperformed that with triangular MFs, the differences between these two cases were insignificant. Therefore, only the IT2 VSL with Gaussian MFs is presented in the rest of the paper. It is to be noted that the IT2 cases yielded MTT medians close to that of their detector-based counterpart when 10% or more PVs were present.

**Figure 8:**
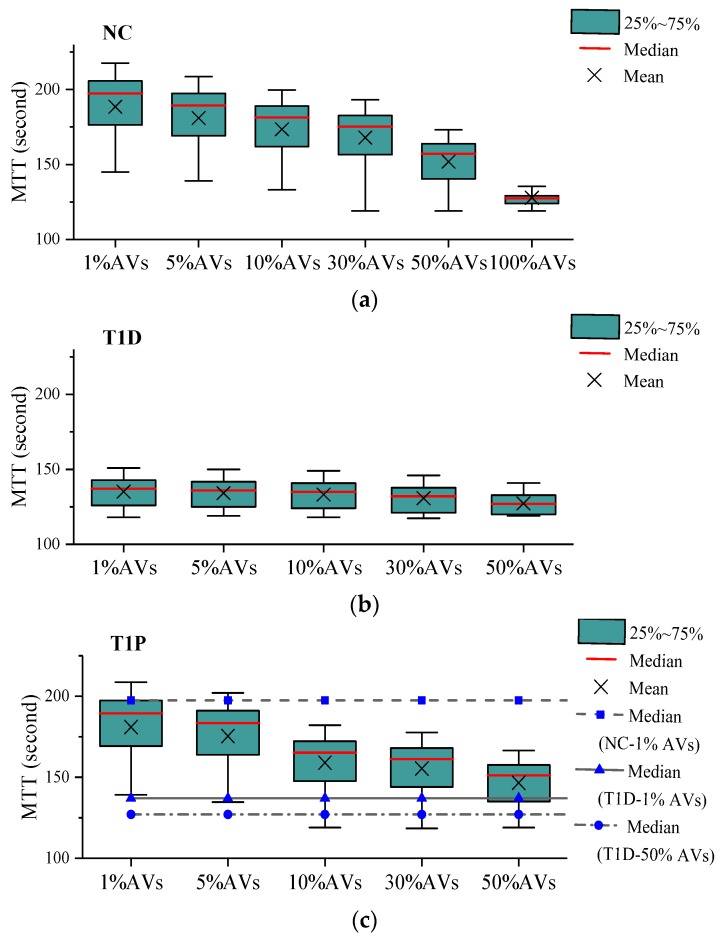

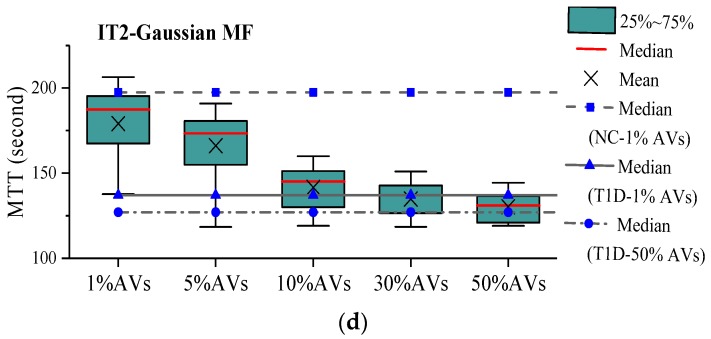
Boxplots of MTT values (the network equipped with CAVs): (**a**) NC&T1D scenario, (**b**) T1P scenario, (**c**) IT2-Triangular MF scenario, (**d**) IT2-Gaussion MF scenario.

Figure 8 summarizes 10-day MTT values for the network equipped with CAVs. Remarkable MTT reduction was witnessed when a large number of CAVs were deployed. Due to traffic amelioration, the VSL was not even triggered at all at 100% CAV penetration rate. The main reason for this might be that CAVs have smaller headways and consequently lead to higher maximum-allowed-volume (capacity). Again, the VSL using detector data recorded the best performance among all the examined scenarios. Nevertheless, the proposed CAV-based VSL was comparable to its detector-based counterpart when sufficient CAVs (>10%) were available.

## 6. Conclusions

Fixed-point sensors are often associated with high installation and maintenance costs. There is a need to develop “infrastructure-free” control systems that can be deployed for jurisdictions with a lack of resources for installation of traffic monitoring infrastructure. To achieve such a goal, this research explored the utilization of CAVs in VSL control systems. The presented VSL system employs CAVs to collect traffic information and requires no fixed-point sensor at all. The performance of the presented VSL system was examined using a real freeway section located in Auckland, New Zealand. The simulation results demonstrate that when more 10% CAVs are deployed, the performance of the proposed VSL system can approach that of the detector-based system. However, it was found that CAV deployment may make VSLs obsolete at very high CAV-equipment rates. While maybe a bit too optimistic, it has been expected that about 70% of all vehicles will have some level of autonomy (Levels 1–3) before fully automated vehicles (Level 5) become commercially available in 2025 [31]. The market share of connected cars is estimated to reach a 100% penetration rate by 2026. Compared to this estimation, the projection for the penetration rate of Level 5 AVs is not as fast, which is expected to reach 25% by 2030 [32]. It is noted that these results cannot be generalized as they are based on a particular section of the Auckland motorway modelled in the SUMO micro-simulator. The model can have its own limitations to represent real-world traffic conditions. It is recommended to conduct similar investigations under a range of different traffic conditions and for a range of motorway networks to verify and then generalize any such results. 

## Figures and Tables

**Table 1:** Rule-base for IT2 FLS based VSL.

No.	Weight	Premise	Consequent
**1**	*w*_1_	IF density *k* is low (*A_L_*)	THEN speed limit *S* is extremely high (*Y_EH_*), *Y_EH_* = [${\underline{y}}_{EH},\;{\bar{y}}_{EH}]$
**2**	*w*_2_	IF density *k* is medium (*A_M_*)	THEN speed limit *S* is high (*Y_H_*), *Y_H_* = [${\underline{y}}_{H},\;{\bar{y}}_{H}]$
**3**	*w*_3_	IF density *k* is high (*A_H_*)	THEN speed limit *S* is medium (*Y_M_*), *Y_H_* = [${\underline{y}}_{M},\;{\bar{y}}_{M}]$
**4**	*w*_4_	IF density *k* is extremely high (*A_EH_*)	THEN speed limit *S* is low (*Y_L_*), *Y_H_* = [${\underline{y}}_{L},\;{\bar{y}}_{L}]$
